# The 3-D Skills Model: a Randomised Controlled Pilot Study Comparing a Novel 1–1 Near-Peer Teaching Model to a Formative OSCE with Self-regulated Practice

**DOI:** 10.1007/s40670-021-01369-w

**Published:** 2021-09-01

**Authors:** C. Robertson, Z. Al-Moasseb, Z. Noonan, J. G. Boyle

**Affiliations:** 1 NHS Greater Glasgow and Clyde, Glasgow, Scotland; 2 Undergraduate Medical School, The University of Glasgow, Glasgow, Scotland; 3 Anaesthetics Department, Queen Elizabeth University Hospital, 1345 Govan Road, Glasgow, G51 4TF Scotland; 4 Glasgow Royal Infirmary, NHS Greater Glasgow and Clyde, Glasgow, Scotland

**Keywords:** Near-peer, Formative assessment, OSCE, 3-D Skills Model, Medical education

## Abstract

**Introduction:**

Near-peer teaching is a popular pedagogical teaching tool, with well-recognised benefits for students and tutors. There are multiple existing models to structure these interventions, but it is often unclear how they translate to academic attainment. We designed a novel near-peer teaching model that expands on previous research.

**Methods:**

Our model was piloted in a formative Objective Structured Clinical Examination (OSCE) setting, trialled on 22 pre-clinical medical students to establish feasibility, acceptability and descriptive outcomes that could inform the design of a larger study. Students were randomly assigned to intervention or control cohorts. Each cohort undertook 5 min formative OSCE assessments with either 3 additional minutes of structured teaching or 3 min of self-regulated practice before reattempting the first OSCE station. Checklist marking sheets for 1^st^ and 2^nd^ sittings were collected by independent external markers, in addition to a global assessment rating in which we used the Borderline Regression Method to establish the station pass mark.

**Results:**

A quantitative and qualitative result analysis was performed, demonstrating that students gained on average 3 additional marks after teaching with this model. Students and student-tutors reported increased confidence, high course satisfaction and evidence of reflective practice.

**Discussion:**

We established acceptability and feasibility outcomes. The descriptive outcomes will support the design of a larger, adequately powered study required to demonstrate translation to summative exam performance.

**Supplementary Information:**

The online version contains supplementary material available at 10.1007/s40670-021-01369-w.

## Introduction

Near-peer teaching (NPT) as a pedagogical teaching method promotes effective learning through constructivist education theory with goal-orientated learning outcomes thought to be established through cognitive congruence [1–3]. Cognitive congruence describes the experience proximity of a near-peer tutor which contributes to effective learning perhaps due to heightened awareness of learner capability [2, 3]. The concept stems from Vygotsky’s work on the ‘zone of proximal development’, a branch of scaffolded learning where tasks are calibrated to the learner’s level in order for them to solve a problem guided by a senior [4]. Near-peer tutors may recognise the zone of proximal development better than content experts due to their relatively recent similar learning experiences [1]. This potentially enables better content processing for students during the learning events [2, 3]. Learning achieved in near-peer-delivered events is mutual; students gain knowledge, and tutors themselves consolidate their own knowledge, and cultivate transferable professional skills [5].

Advantages of NPT quoted in literature include its minimal cost and unique benefits to students and tutors [6, 7]. However, scepticism exists on the quality of educational attainment compared with faculty tuition with concerns about the fidelity of NPT interventions and competence of student-tutors [5, 8–10]. Additional tuition or teaching aids can be used to improve the effectiveness of NPT [6].

Studies in current literature from NPT interventions are limited, focusing on student reaction and rarely assess translation to summative success [6, 8]. Such examples use Peyton’s 4-step approach for tuition or formative adaptions of summative examinations [2, 5, 11, 12]. Peyton’s 4-step approach is a well-recognised model for teaching psychomotor skills where the tutor demonstrates a skill, deconstructs the procedure, checks student comprehension and then allows the student to perform the skill [11]. Whilst Peyton’s approach has been well researched in academia, it is a time-intensive model that may have reduced practicality, for example, in a clinical setting [11].

An alternative is the formative Objective Structure Clinical Examination (OSCE) [2, 5]. OSCEs are a widely used clinical assessment tool, evaluating the attainment of competency in a variety of clinical practices and procedures. Formative adaptations replicate the fidelity of summative OSCEs, offer a safe learning environment and improve student confidence in examination skills [2, 5, 9]. The educational principle behind OSCE as a formative assessment tool utilises a constructivist approach with feedback and reflection fostering the acquisition of competency [13]. This utilises Knowles principles of andragogy, suggesting experience is paramount in driving adult learning [14]. Kolb’s experiential learning cycle also supports educational attainment in OSCEs [15]. This reflective education model is frequently used in simulation training where students actively reflect on a ‘concrete experience’, i.e. their performance in an OSCE, and use this to direct future learning [16]. However, despite the theoretical framework underpinning a rationale for formative OSCE use, there is conflicting evidence regarding the actual educational attainment from a formative OSCE [5, 17, 18]. Many studies have been unable to show objective performance improvement [5, 8, 17, 18]. Some studies have suggested that self-regulated practice or student-tutor feedback can enhance formative OSCEs to provide a more tangible learning benefit [5, 8, 19].

Concise learner-centred approaches to teaching have been popularised recently with models such as the five-step Micro Skills Teaching design (One-Minute Preceptor) [12]. These have particular advantages in time-pressured environments and drive critical reasoning skills [12, 20]. Such a design would have advantages over Peyton’s and formative examination models by addressing both the educational needs of the learner and logistical practicalities. However, a micro-skills equivalent for psychomotor skill attainment has not been reviewed in the literature.

Using these three aforementioned models (Peyton’s 4-step approach, micro-skills teaching and a formative OSCE), as a basis for our NPT model, we designed a novel hybrid teaching approach that would be reproducible in a variety of clinical and non-clinical settings. We were keen to demonstrate evidence of educational attainment from our NPT intervention. Additionally, we stipulated that our NPT model should be easily taught to and delivered by near-peer tutors, including medical students. To demonstrate the feasibility of our new model, termed the 3-step Deconstructed Skill (3-D Skill) model, we conducted a pilot study to establish acceptability and descriptive outcomes that could inform the design of a larger study.

To establish a control, we aimed to compare the 3-D Skill model to the formative OSCE model [5], a pre-existing and well-established clinical skills teaching modality at our school. All students would sit the same formative OSCE station for comparison. The control group would then have time for self-regulated examination practice, and the intervention group would have 3-D skills teaching incorporated into the station time, enabling all students to have the same overall time at each OSCE station. OSCE performance would be assessed by external markers utilising checklist marking sheets and a global assessment rating.

The primary aim was to identify if there was an improvement in student checklist scores after 3-D skills teaching to support the design of a larger study. Secondary aims were to examine qualitative data to establish the acceptability of the 3-D skills teaching.

## Methods

### Designing the Model

**Fig. 1:**
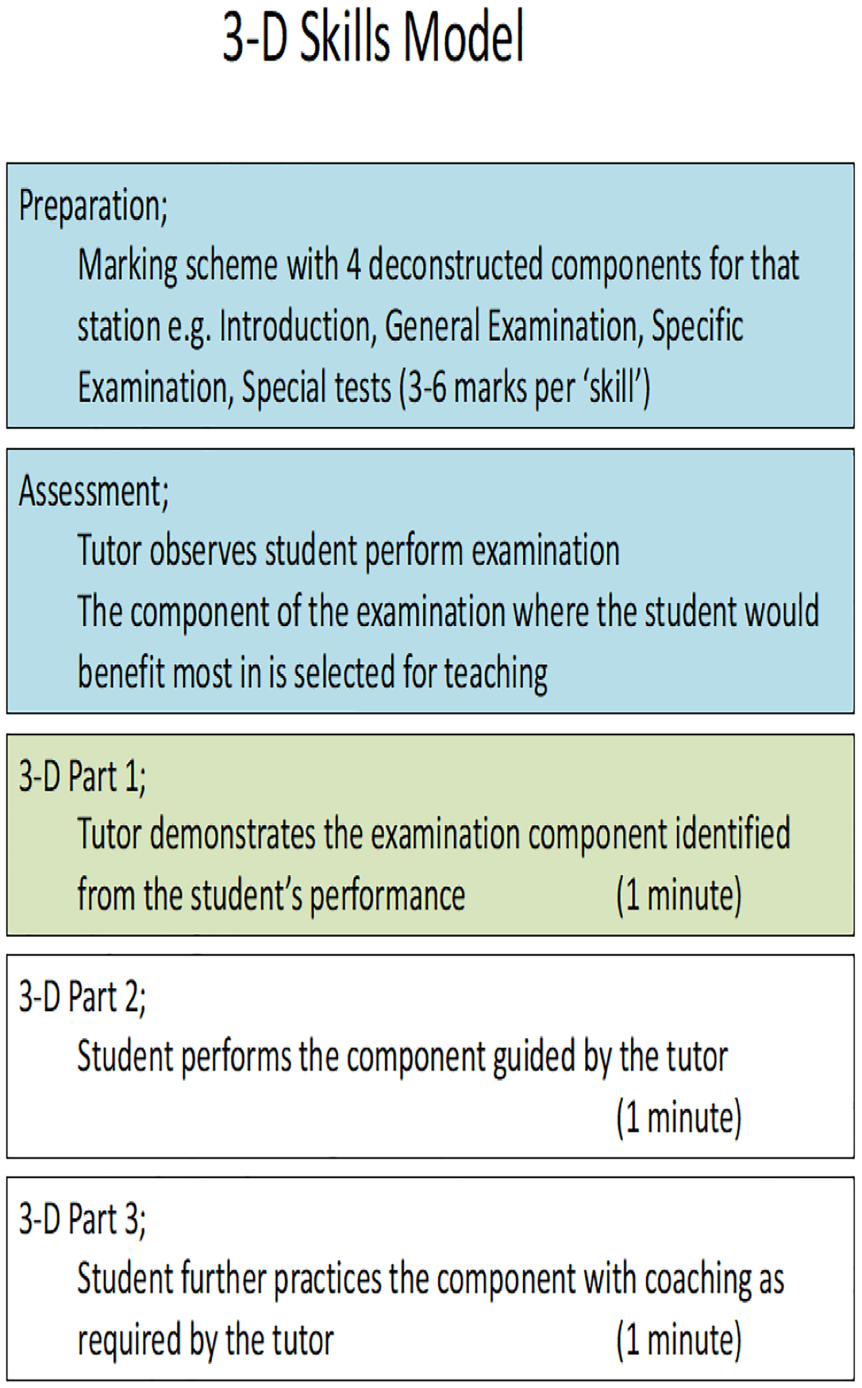
The 3-D skills model adapted to a formative OSCE

The 3-D skill model, shown in Fig. 1, combined aspects from Peyton’s 4-step approach, micro-skills teaching and formative OSCEs [5, 11, 12]. Previous research suggests that the deconstruction and comprehension stages of Peyton’s 4-step approach constitute the greatest learning gain [11]. These two steps were structured around a formative assessment, focusing teaching to an area where the student would benefit most. This utilises a learner-centred approach, as seen in the micro-skills teaching models with the 1 min preceptor [10]. NPT is limited to 3 min in duration, with the tutor identifying one aspect to focus on using learning aids. For example, in a cardiovascular examination, the tutor may choose to focus on the precordial examination. A worked example of how this ran in practice is described in Appendix 1. During the first minute of the 3-D skills model, students observe a demonstration of the targeted clinical examination component (similar to the deconstruction stage from Peyton’s 4-step approach) [11]. The second and third minutes of the 3-D skills model reflect the ‘comprehension stage’ allowing the student to continually practice the component, guided by the examiner, to reinforce successful acquisition of the targeted learning outcome.

#### Curriculum and Assessment Strategy

All work was completed on campus at the University of Glasgow medical school which has a 5-year MBChB course. The first 2 years are pre-clinical where students are introduced to clinical communication and clinical examination skills. These are subsequently assessed throughout their training with formative and summative OSCEs in years 2, 3, 4 and 5. OSCEs at Glasgow utilise checklist marking schemata, based on standardised domain-based mark allocation in combination with a global rating scale which is used to determine the station pass mark. Comprehensive written descriptions of on what constitutes a ‘borderline candidate’ for each year group are provided.

### Student-Tutor Education

**Fig. 2:**
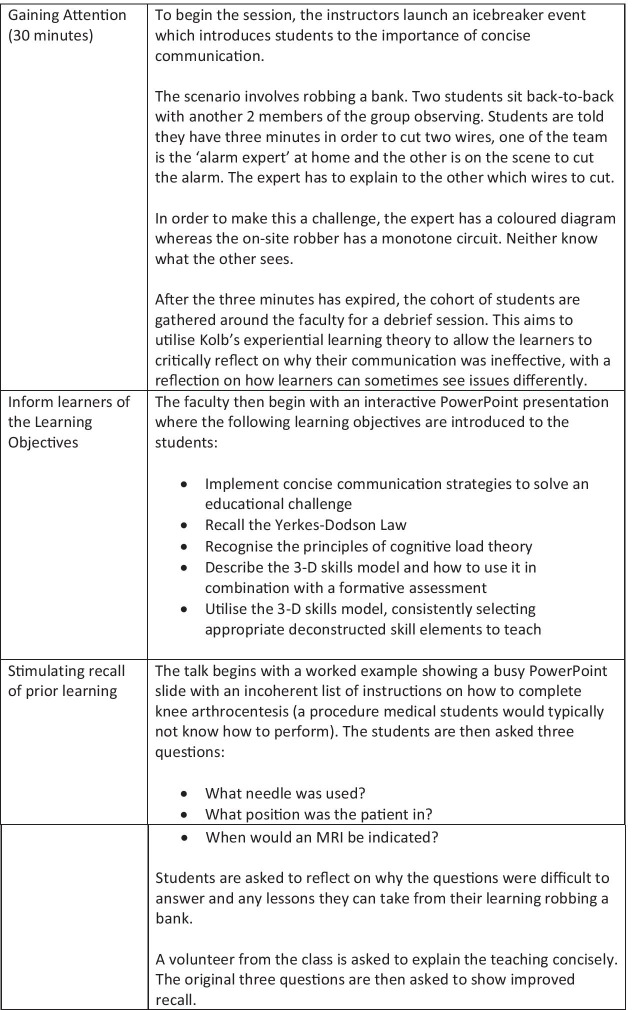

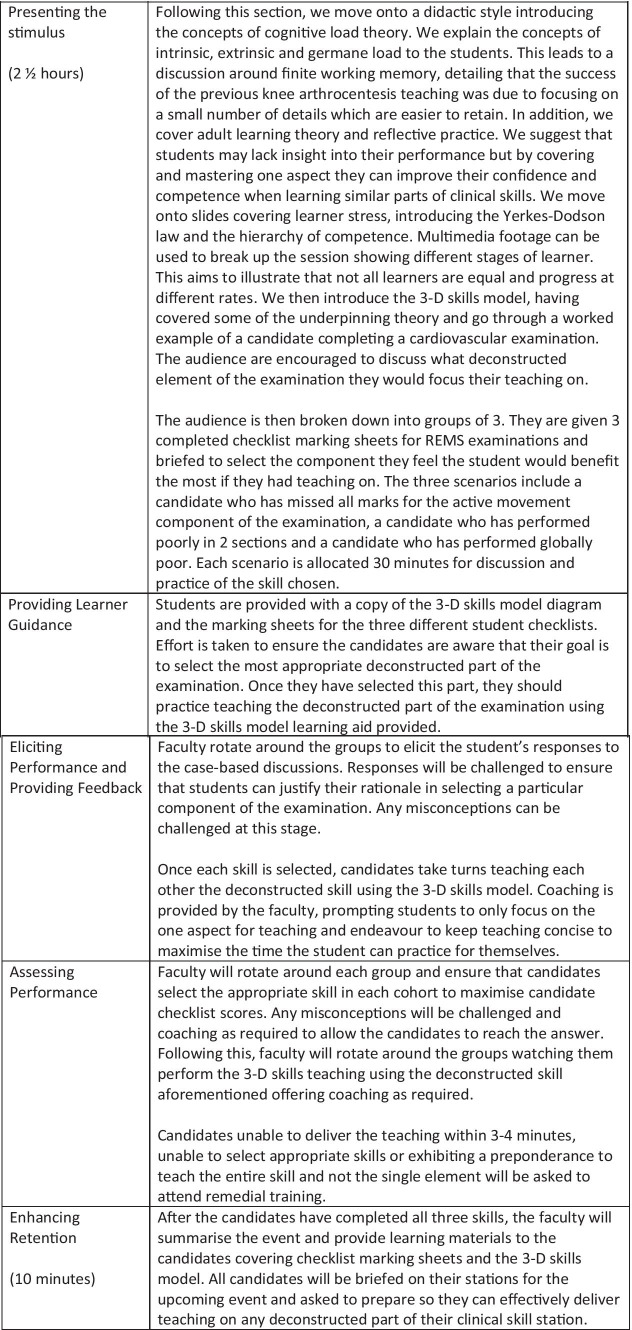
Student tutor education event

Student-tutors, in their 4^th^ year or above, were recruited from the medical school via social media. They were invited to attend a near-peer half-day clinical teaching session run by the team of junior doctors who designed the 3-D skills model. The event timeline is shown in Fig. 2 using Gagne’s nine steps for instructional design [21]. This session introduced the learner to some of the fundamental theory underpinning the 3-D skills model and allowed an assessment of competence prior to implementing the new teaching strategy. Twenty-two students attended this training session; we required twenty of these students to attend the subsequent formative OSCE, allowing a redundancy pool to cover absenteeism.

### Study Design

We conducted a formative OSCE for second-year pre-clinical medical students utilising 5 min clinical examination stations covering their core curriculum. Our aim was to provide an authentic representation of the summative OSCE experience for students, which previous research has suggested may reduce student anxiety in subsequent assessments [5]. The OSCE station structure and materials were adapted from the summative examination format, offering comparable validity. Year 2 students were invited to attend the learning event in 2017 via campus emails and social media. The study was undertaken in the clinical skills suite on campus at the University of Glasgow. Approval for the study was gained from the University of Glasgow ethics committee.

On arrival, students were randomly allocated to the intervention or control cohort via a random number generator. Care was taken to ensure students were not informed which cohort they were allocated to. Students were briefed in separate rooms and kept apart until the conclusion of the study. The student-tutors were also briefed in separate rooms for intervention and control cohorts respectively. Two student-tutors were allocated per station where they remained until the study concluded with no crossover between the intervention and control tutors. Students were exclusively rotated around an OSCE circuit of single-room stations, guided by faculty facilitators. To assess fidelity of the intervention and control, the faculty approached student-tutors at station changeovers to observe if they were following the brief. Examination conditions were enforced to maintain authentic examination conditions and prevent students from discussing the stations.

We utilised external markers (foundation year 2 doctors or above) to formally mark the second-year students with one allocated per station. They received training on how to complete the checklist marking sheets using the same formal guidance the University provides to summative examiners. This constituted a written summary and training video produced by experienced University faculty. They defined the MBChB2 borderline pass candidate: ‘With regards to examination technique, the MBChB2 borderline student will be aware of the fundamental approach to clinical examination but may omit sections of examination or lack structure in their approach to this’. The external markers were independent, not involved in the teaching delivered by the 3-D skills tutors and not involved in the research design. Like the student-tutors, they were also kept in isolation and they were not briefed on the differences between the two cohorts. Utilising external markers to formally score the students allowed the 3-D skills tutors to focus on their teaching role in addition to improving assessment validity.

**Fig. 3:**
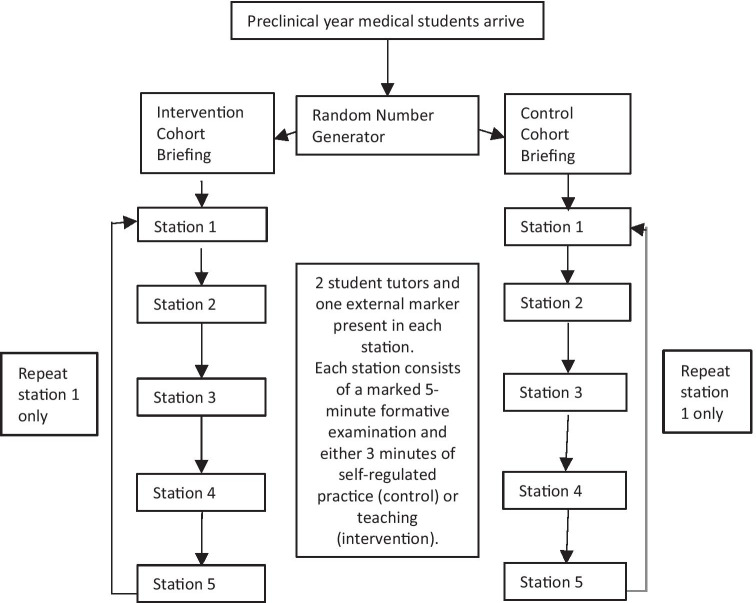
Timeline of formative OSCE event

Each station consisted of a formative examination featuring a 5 min assessment utilising a checklist marking sheet marked by the station examiner. This was followed by 3 min of intervention (3-D skills teaching by tutor) or control (student self-regulated examination practice) for a total of five stations. To enable us to assess the effects of the intervention, all students then re-sat their first station, the cardiovascular examination station, after completion of the event. Both student cohorts ran in parallel at opposite ends of the clinical skills suite. The student-tutors took alternating roles as volunteer patient or tutor/second examiner for each candidate. A timeline of the event is shown in Fig. 3. A tannoid system was used to signal the start of the clinical station, the end of the 5 min assessment period and the end of the station. This ensured that the 3 min allocated intervention/control time was followed by all candidates.

#### Checklist Marking Sheets

Checklist marking sheets were sourced from previous OSCEs used within the medical school, written by faculty and subject experts suggesting appropriate construct validity. Each checklist constituted twenty items arranged in a binary (done/not done) scoring system. The global rating scale was included, to align the score sheet with those used in authentic summative assessment marking at Glasgow. Utilising a binary checklist marking sheet with global assessment served as a means to assess concurrent validity, as our construct closely followed the established summative examinations. The examination comprised five stations: abdominal examination, cardiovascular examination, upper neurological examination, knee joint examination and respiratory examination assessing a range of content to optimise validity.

As shown in the sample checklist in Appendix, the criterion assessed in an OSCE is typically at the ‘shows how’ level of Miller’s pyramid [22]. The OSCE style demonstrated as a binary does/does not perform would imply high construct validity when assessing these skills. To account for the potential contention of our global-scale assessment in contrast to faculty, we utilised the Borderline Regression Method, a reliable standard setting tool commonly used to assess OSCEs, to remove inference from inter-rater reliability [23]. In addition, as the control repeated the final station, we have a measure of test–retest reliability. These measures address validity and reliability of our OSCE assessment and evidence consideration of Messick’s validity framework, seen as a standard when assessing the evidence to support a test [24, 25].

### Commonalities to Both Cohorts

Both student cohorts received identical briefs. This brief included detail about the examination logistics that each station would comprise a 5 min formative OSCE station and that there would be 3 min of practice to go over any part of the examination. Students were told that the 3-min practice time may or may not be guided by one of the examiners. Both cohorts sat identical clinical scenarios.

### Control Cohort

The control cohort featured two student-tutors in each station, ten in total. These student-tutors would alternate roles of patient or tutor that would provide a prompt about the self-regulated practice if required. During the brief for the control cohort, the student-tutors were instructed that the students would be given 5 min for a marked formative OSCE followed by 3 min of self-regulated practice on the simulated patient. If a student required a prompt, the student-tutors were to inform them that they had;Three minutes to practice any part of the examination again’. These control tutors were informed not to provide any teaching or feedback to the student during this time. Control tutors were briefed separately from the intervention cohort and there was no crossover between groups.

### Intervention Cohort

The intervention cohort featured two student-tutors per station, ten in total. These tutors alternated roles of patient or tutor that provided the 3-D skills teaching. The brief for these student-tutors was to deliver 3-D skills teaching after the 5 min formative examination. Learning aids were provided, and a short recap of the 3-D skills model delivered by event faculty during the brief.

### Data Collection

To demonstrate quantitative results, we asked our external markers to complete checklist marking sheets for each student, allowing statistical comparison of the first and second sittings of the cardiovascular 5 min formative examination. This included a global assessment, for which we would use the Borderline Regression Method to establish an approximate pass mark for the station [23]. For qualitative data, we asked students to complete feedback sheets before and after the event. The pre-event feedback sheet consisted of five questions that included demographical information, asking students to self-identify age group, gender, attendance at previous peer teaching events and post-graduate status. In addition, a 10-point Likert scale was used to identify student confidence level to pass the summative OSCE. Our post-event feedback sheet consisted of a 5-point Likert scale question for event satisfaction, a 10-point Likert scale to identify student confidence to pass the summative OSCE and three free-text questions prompted to identify aspects of the event that were useful and could be improved and any other comments from the candidate. These free-text comments were subsequently analysed using thematic analysis described below [26, 27].

We asked our student-tutors to complete a feedback sheet after the student-tutor education session. This featured a 5-point Likert scale analysis for event satisfaction, a 10-point Likert scale for confidence to deliver clinical skills teaching after this event and a prompted question to discuss if they felt 3 min was sufficient time to teach. In addition, student-tutors completed a feedback sheet after the formative OSCE event which featured a 5-point Likert scale analysis for event satisfaction and three free-text questions prompted to identify aspects of the event that were useful and could be improved and any additional comments.

### Thematic Analysis

Thematic analysis was completed using Braun and Clarke’s approach [27]. This involved coding free-text comments and developing themes that best matched patterns across the various student comments. A consensus approach was taken by the two primary researchers to produce the final structure of themes and respective comments.

#### Quantitative Analysis

This small-scale trial uses a subset of the student population with the assumption it reflects the year group. Student confidence levels and checklist marking sheets will be assessed using the sample mean and 95% confidence intervals, respecting that they will likely be underpowered for meaningful quantitative statistical analysis. The student event satisfaction will be assessed by comparing the median value for the two cohorts.

## Results

We recruited twenty-two second-year medical students with eleven randomly allocated to control and intervention cohorts respectively.

### Demographics

The intervention cohort had four male and seven female students in comparison to the control with five male and six female students. The intervention cohort had six home/European students compared to seven in the control, with the remainder non-European students. There were no post-graduate students in either cohort. Both cohorts had seven students who had previously attended a peer/near-peer clinical skill teaching event in the past year. This sub-section represented approximately 10% of the year group. In the year group population, 60% of students identified as female and 12% of students were from overseas.

### Checklist Scores

Intervention candidates increased their mean score on the cardiovascular station from 11.0 (95% CI [8.8, 13.2]) to 14.3 (95% CI [12.1, 16.5]), corresponding to a mean increase of 3.3, as shown in Table 1. The control cohort candidates’ mean checklist score increased from 10.9 (95% CI [8.5, 13.3]) to 11.1 (95% CI [8.7, 13.5]), an increase of 0.2.

**Table 1:** Descriptive statistical data for checklist score, event satisfaction and confidence levels

	Mean	Median	Standard deviation	Standard error of the mean
Candidate checklist scores				
Intervention cohort 1^st^ sitting checklist scores	11.0	12	3.8	1.1
Intervention cohort 2^nd^ sitting checklist scores	14.3	16	3.5	1.1
Control cohort 1^st^ sitting checklist scores	10.9	11	3.9	1.2
Control cohort 2^nd^ sitting checklist scores	11.1	11.5	3.9	1.2
Student event satisfaction				
Intervention cohort	4.9	5.0	0.3	0.1
Control cohort	4.0	4.0	0.4	0.1
Student confidence levels				
Intervention cohort pre-event	3.8	3	1.6	0.5
Intervention cohort post-event	7.2	7	1.4	0.4
Control cohort pre-event	3.6	3	1.9	0.6
Control cohort post-event	6.4	6	2.2	0.7

### Global Rating

**Fig. 4:**
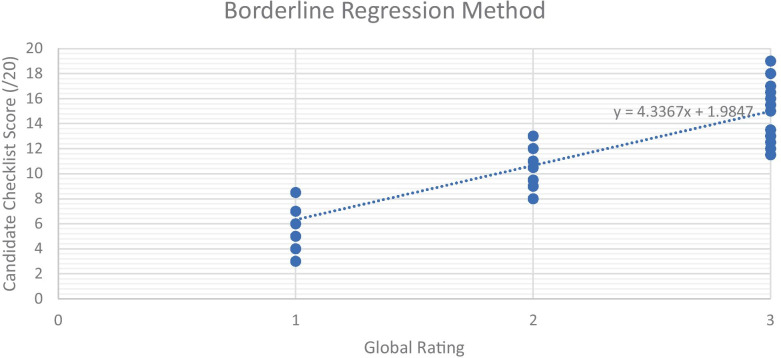
Borderline Regression Method setting candidate pass mark

Four of the eleven students in the control cohort were awarded a pass on the first sitting, three were awarded a borderline pass and four received a global failure rating. In contrast, three of the eleven students in the intervention cohort received a pass on first sitting, four received a borderline pass and four received a failure rating. On resit, one student in the control cohort improved their global rating from borderline pass to pass. In the intervention cohort, the four candidates who received fail grades were awarded a borderline pass and three of the four borderline pass candidates received a pass on the global assessment. To account for inter-rater variability, all 44 global ratings with corresponding checklist scores (first and second sittings) were plotted on a scatter graph and a regression line plotted as shown in Fig. 4. The corresponding pass mark was 10.6. Using the borderline regression method, nine out of the eleven intervention candidates would have passed whilst six of the eleven control candidates would have achieved a pass on second sitting.

### Event Satisfaction

The rating form featured a Likert scale ranging from 1 to 5 with 1 representing a poor event and 5 representing an excellent event. The intervention cohort achieved a median satisfaction rate of 5.0 compared to 4.0 in the control cohort as shown in Table 1.

### Confidence Levels

The control cohort increased their mean confidence from 3.6 (95% CI [2.4, 4.8]) pre-event to 6.4 (95% CI [5.0, 7.8]) post-event, an increase of 2.8 as shown in Table 1. In comparison, the intervention cohort achieved a mean confidence increase of 3.4. Their mean pre-event confidence level was 3.8 (95% CI [2.8, 4.8]) compared to 7.2 (95% CI [6.4, 8.0]) post-event.

### Qualitative Data

#### Faculty Troubleshooting

At the intervention cohort briefing, some tutors queried how to approach a candidate who was performing globally poorly where there may have been several teaching areas to cover. Tutors were reassured to focus on only the one part of the examination they identified from the formative assessment and complete the 3-D skills approach accordingly. Faculty offered troubleshooting advice whilst assessing fidelity of the intervention/control at station changeovers. There were two recurring themes highlighted by event faculty for each cohort. The most frequent queries regarded supply of stationary, including pens, stethoscopes and blank paper for notes, and logistics of the night. Three tutors in the intervention cohort queried how to approach a candidate who was performing globally poorly and in whom there may have been several teaching areas to cover. On two instances, tutors in the intervention cohort admitted they attempted to cover an additional part of the examination after the candidate had practised the skill but ran out of time. These tutors were reminded to let the candidate repeatedly practice the part of the examination using step 3 of the 3-D skills model, after completing steps 1 and 2, and not pursue an additional area. Regarding the control cohort, student-tutors reported anxiety with two students who performed poorly and were perceived to be ineffective at self-regulated practice. Tutors were reminded to allow the students to practice unaided for the 3 min and provide a prompt to the candidate if required to encourage them to practice as described in the tutor brief. Faculty ensured that candidates left the examination room when the tannoid concluded the station.

#### Students

Table 2 shows the free-text comments for the intervention and control cohorts. Key themes were identified after coding individual comments. All eleven students in the intervention cohort contributed at least one comment. Most (10/11) students in the control cohort wrote at least one free-text comment. Common themes identified from both groups included reflection for future learning, students valuing experience of a formative examination and event satisfaction. An additional theme arose for the intervention cohort featuring a positive response to concise teaching.

**Table 2:** Student free text answers with thematic analysis

Key themes	Intervention cohort comments	Control cohort comments
Reflection for future learning	‘Gave me an good idea of how much work I need to do’	‘Got an idea of how much I don’t know’
	‘… good to see where I am losing marks, so I know what to go over’	‘I felt a bit clueless when attempting some stations’
Event satisfaction	‘Feedback and OSCE practice was great’	‘…I found it very helpful to go over things’
	‘…fantastic, well taught and very helpful’’	‘Good to practice the stuff I’d covered only once’
	‘The 1–2-1 teaching in a low-pressure environment was great. Thanks very much for organising this!’	‘Fantastic and very helpful, thank you very much’
	‘…Loved the way it was taught’	
	‘…lovely attitude in general’	
	‘1–1 teaching was great!’	
Students value fidelity of formative examination	‘Great that it’s in exam conditions’	‘I liked the realistic timings’
	‘Properly structured OSCE with realistic timings’	‘…get an idea of procedures’
	‘The teaching after made the timings not like the real examination’	‘OSCE style was great’
		‘Simulated the feel of the real OSCEs’
Positive response to concise teaching	‘Teaching was very concise and informative’	
	‘…very simple to understand and remember’	
	‘…I like that it was straight to the point’	
	‘… made it very easy to remember’	

#### Student-Tutors

The event satisfaction score from the student-tutors was a median of 5 with all but one rating the event as 5/5 (excellent). Of the twenty-two student-tutors who attended the half-day skills session, seventeen rated their confidence to teach as 10/10, with the remaining five rating 8/10 or higher (mean 9.7). Most (19/22) student-tutors felt that 3 min was sufficient time to teach with one suggesting it was too long and two student-tutors suggesting it was too short. Free-text themes are highlighted in Table 3. Themes included improving teaching confidence and consolidating prior knowledge.

**Table 3:** Student tutor feedback sheet analysis

Key theme	Comments
Consolidated knowledge	‘Great revision for my exams’
	‘Keep my skills up to date’
Improved teaching confidence	‘The teaching skills worked well’
	‘I felt more comfortable knowing what to say’
	‘The model and checklists made this easier’

## Discussion

Students who experienced the 3-D skills model showed an average of 3.3 checklist score increase on second sitting (out of 20). In contrast, the average increase in the control cohort was considerably smaller, 0.2. Despite the small numbers in this study, the potential to increase a checklist score by three marks could be significant in determining the difference between station pass or fail for borderline candidates in summative examinations. This is demonstrated when analysing the students global rating as assessed by the external markers. We show that an additional three (27%) of the students in the intervention cohort achieved a pass in comparison to the control cohort, despite a comparable baseline. All candidates in the 3-D skills cohort were able to attain additional marks from the targeted area of teaching from their first sitting through to their second sitting. This would suggest that the teaching is successfully delivered and effectively retained, at least in this short time frame. In addition, it would imply that the 3-D skills model is a feasible education tool to be used in conjunction with a formative examination.

Student-tutors rated the 3-D skills model positively and most felt that 3 min was an acceptable amount of teaching time. Considering the range of candidate preparation as evidenced by first sitting checklist scores ‘4–16’, this pilot covers an assortment of different student capabilities. As only two (9%) tutors found the teaching time to be short, we can be encouraged that 3 min is feasible to deliver this intervention in a larger study. In addition, achieving an increased checklist score during this time suggests that the intervention model can be effectively delivered to student-tutors in a half-day teaching programme. Thus, training is not labour intensive suggesting feasible upscaling when recruiting additional tutors.

Fidelity of this intervention was partially assessed by short informal debrief interviews by faculty at station changeovers. Despite a wish to teach further, there were no clear breaches of protocol identified. A more invasive assessment of fidelity may have compromised the integrity of the external examiners and subject the study to increased bias. However, in hindsight, we could record the teaching sessions in each room. Subsequent playback could be used to ensure all sessions were in keeping with the fidelity of the study and should be considered for a larger study. The control showed similar fidelity as assessed by the short debriefs. However, the student-tutor anxiety associated with withholding teaching to poor performing candidates would suggest that caution is needed when upscaling the event to ensure that tutors do not deviate from protocol by providing teaching.

The issues raised by student-tutors during these debriefs were of interest, particularly how to approach a candidate performing poorly in all sections. In these candidates, an additional three marks (15%) would be unlikely to correlate to examination success alone and perhaps there is futility selecting one area. Indeed, an improvement in metacognitive and affective behaviours would likely be more beneficial to develop globally improved self-practice and educational processes [28, 29]. Future studies could illustrate long-term metacognitive and affective changes particularly in students with prior ineffective learning strategies. Perhaps this is achieved through revised goal orientation, using the near-peer tutor as a role model [3]. In addition, achieving effective learning of a single focus area may foster self-confidence, further developing self-regulated learning out with the formative examination experience [30].

A challenge to the feasibility of this design is reliance on voluntary attendance. Initial study designs were to repeat all stations after teaching, but this had low acceptability when pitched to near-peer tutors. The design aimed to optimise student-tutor time commitment and cover a range of subjects for student satisfaction whilst still offering a meaningful data source for comparison. One control cohort student commented that they wanted additional examination stations suggesting there is an optimum number to maintain course satisfaction. Despite the logistical challenges of providing out of hours education, the same design could be repeated on multiple dates to increase capacity. Certainly, the demand for this teaching session and the student-tutor education course was high. We achieved our target numbers within 24 h of advertising, supporting the upscale of this project.

We demonstrate high levels of student satisfaction with this NPT intervention, in keeping with existing literature [5, 10, 31]. The 3-D skills cohort achieved a higher satisfaction rate than the control, which suggests non-inferiority of the design. Both cohorts showed an increase in baseline confidence which is also in keeping with existing literature [5, 10, 31]. Of interest, this improvement in confidence was similar in both cohorts yet the correlation to increase in checklist scores was markedly different. This would highlight a potential weakness in previous studies relying on confidence alone to demonstrate validity [8]. Indeed, studies have looked at overconfidence which may be a factor in students performing negatively in summative assessments [32].

Most students provided free-text comments, which was highly informative. One student commented that they would prefer teaching after the event rather than integrated during it. This is an interesting consideration that would likely increase the fidelity of the event, mimicking a summative examination, but perhaps detract from the educational attainment. Benefits of near-peer teaching, including the sense of realism and safety, were mirrored in both the intervention and control student comments [5]. Feedback was almost ubiquitously positive, further supporting the high student satisfaction rates and overall acceptability of this study. Two of the students in the intervention cohort showed evidence of reflecting on this experience and how it might impact their study behaviour in the future which could demonstrate affective domain learning [8]. Perhaps focusing on a small aspect of the examination encourages students to reflect upon areas of weakness in their own clinical skills practice. Generating affective behaviours, valuing the teaching input and encouraging metacognition may allow attainment of long-term outcomes and perhaps translate to summative examination success [8, 33].

As we based the 3-D skills model on the existing literature [5, 10, 31], we anticipated a favourable student reaction in terms of student satisfaction and confidence levels. A previous study suggested that the essential components for a Peyton’s 4-step approach are the ‘deconstruction’ and ‘comprehension steps’, supporting their focus in our model [11]. In addition, by keeping the teaching concise and focused, we derive some of the benefits highlighted by the learner-centred One-Minute Preceptor model [12]. Indeed, reducing the complexity, or intrinsic load, may be better received by students whose short-term working memory is likely already challenged by the formative examination [34]. This may explain why this information is well retained by students as assessed by the improvement in their checklist rating post intervention.

Both cohorts feature a comparable demographical mix of home and non-European students. The two cohorts additionally showed a similar baseline performance in first sitting checklist score, global rating assessment and baseline confidence levels. This suggests that the randomisation was successful in achieving an adequate control. Undoubtedly, the small sample size is a limiting factor when considering the impact of this new model. There is the potential for selection bias based on students who chose to attend the teaching event, although efforts were taken to minimise this including randomisation and validation by comparable baseline and demographic data. Blinding was used to prevent any effect from crossover bias or tutor bias affecting the end result. In addition, utilising independent markers further reduced the impact of bias and affords credibility in the reproducibility of this study. Indeed, some studies are unclear on who marks the checklist scores in these formative assessments which question their credibility in providing a reliable assessment [5, 8].

When considering the adequacy of the control, there is the potential for performance bias. If the student felt they performed well, they may not practice effectively during the self-regulated phase. However, in designing this new model purposely to fill a gap in currently available educational tools, there lacks a direct comparator with a similar time commitment. Self-regulated practice offered the nearest control at the time accounting for variables that could confound the results. This offered an opportunity for us to compare the 3-D skills model to the established formative OSCE model. Whilst we appreciate the addition of self-regulated practice may impact the fidelity of the formative OSCE control, the results appeared comparable to the existing research surrounding formative OSCEs and suggests the feasibility of its use in an upscaled study. A third group comparing 3-D skills performance to Peyton’s 4-step approach would be desirable but feasibly challenging to match multiple variables, including education time, and thus was omitted from this pilot study.

We propose a larger study with appropriate power to further explore the short- and long-term outcomes with this model and, additionally, correlate course teaching intervention with subsequent summative OSCE performance. Based on our experience, we envisage the recruitment of students and student-tutors to be easily achievable; however, there may be challenges in recruiting additional external markers. We propose repeating the design over multiple dates to accommodate an increased number of students without an excessive time commitment from tutors or markers. Given the relative cost neutrality of the intervention, in scale-up, we do not anticipate any significant financial challenge.

## Conclusion

The 3-D skills model demonstrates proof of concept in this pilot study achieving an increase in student checklist scores after teaching. Advantages of this model include its concise design allowing its application to different educational settings and its ability to be delivered effectively by near-peer tutors. The model demonstrates positive student feedback and may improve student confidence levels in examination skills. Future work with larger cohorts will determine the long-term outcomes of this model.

## Supplementary Information

Below is the link to the electronic supplementary material.

Supplementary file1 (DOCX 19 KB)
