# Supplementary material for: The 3-D Skills Model: a Randomised Controlled Pilot Study Comparing a Novel 1–1 Near-Peer Teaching Model to a Formative OSCE with Self-regulated Practice
Source: Med Sci Educ. 2021 Sep 1;31(6):1789–801. doi: 10.1007/s40670-021-01369-w (PMC8651955; doi:10.1007/s40670-021-01369-w)
Supplement: Supplementary file 1 — Supplementary file1 (DOCX 19 KB) [file 40670_2021_1369_MOESM1_ESM.docx]

**Cardiovascular Examination – Template**

**Time:** 5 minutes (examination), 3 minutes (tutoring), 1 minute (changeover)

|  | **TASK** | **Marks** | |
| --- | --- | --- | --- |
| 1. | Introduce self, check patient identity and position patient at a 45-degree angle | 0 | 1 |
| 2. | Explanation of examination and gain consent | 0 | 1 |
| 3. | General inspection (general state, equipment by bed – oxygen GTN etc, colour) | 0 | 1 |
| 4. | Inspect hands (cap refill, tobacco staining, xanthomata, clubbing, splinter haem.) | 0 | 1 |
| 5. | Measures radial pulse (rate, rhythm and volume) | 0 | 1 |
| 6. | Inspect face with special emphasis on central cyanosis | 0 | 1 |
| 7. | Assessment of carotid pulse (rate, rhythm) | 0 | 1 |
| 8. | Assessment of jugular venous pressure including hepato-jugular reflex | 0 | 1 |
| 9. | Inspect precordium (scars, pacemakers, visible pulsations) | 0 | 1 |
| 10. | Locate apex beat with right hand (with manoeuvre when necessary) | 0 | 1 |
| 11. | Correctly locate position of apex beat | 0 | 1 |
| 12. | Left parasternal heave and thrills | 0 | 1 |
| 13. | Auscultate precordium in 4 areas with bell and diaphragm: 1 mark per correctly named area  -Mitral  -Tricuspid  -Pulmonary  -Aortic | 0 | 4 |
| 14. | Auscultate Carotids | 0 | 2 |
| 15. | Offer manoeuvres to accentuate murmurs:  -Turn patient on to left side and auscultate in the mitral area (mitral stenosis)  - Sit patient forward and auscultate over the lower left sternal edge in expiration | 0 | 1 |
| 16. | Mention other key areas, appropriate summary and conclusion | 0 | 1 |

Circle: Pass / Borderline Pass  / Fail   Total   marks: _    / 20

**Cardiovascular Examination – Example**

**Time:** 5 minutes (examination), 3 minutes (tutoring), 1 minute (changeover)

|  | **TASK** | **Marks** | |
| --- | --- | --- | --- |
| 1. | Introduce self, check patient identity and position patient at a 45-degree angle | ~~0~~ | 1 |
| 2. | Explanation of examination and gain consent | ~~0~~ | 1 |
| 3. | General inspection (general state, equipment by bed – oxygen GTN etc, colour) | ~~0~~ | 1 |
| 4. | Inspect hands (cap refill, tobacco staining, xanthomata, clubbing, splinter haem.) | ~~0~~ | 1 |
| 5. | Measures radial pulse (rate, rhythm and volume) | ~~0~~ | 1 |
| 6. | Inspect face with special emphasis on central cyanosis | 0 | ~~1~~ |
| 7. | Assessment of carotid pulse (rate, rhythm) | ~~0~~ | 1 |
| 8. | Assessment of jugular venous pressure including hepato-jugular reflex | ~~0~~ | 1 |
| 9. | Inspect precordium (scars, pacemakers, visible pulsations) | ~~0~~ | 1 |
| 10. | Locate apex beat with right hand (with manoeuvre when necessary) | ~~0~~ | 1 |
| 11. | Correctly locate position of apex beat | ~~0~~ | 1 |
| 12. | Left parasternal heave and thrills | 0 | ~~1~~ |
| 13. | Auscultate precordium in 4 areas with bell and diaphragm: 1 mark per correctly named area  -Mitral  -Tricuspid  -Pulmonary  ~~-Aortic~~ | ~~0~~ | ~~4~~  1 |
| 14. | Auscultate Carotids | 0 | ~~1~~ |
| 15. | Offer manoeuvres to accentuate murmurs:  -Turn patient on to left side and auscultate in the mitral area (mitral stenosis)  - Sit patient forward and auscultate over the lower left sternal edge in expiration | ~~0~~ | ~~2~~  1 |
| 16. | Mention other key areas, appropriate summary and conclusion | ~~0~~ | 1 |

Circle: Pass / ~~Borderline Pass  / Fail~~   Total   marks: 13  / 20

This candidate lost marks when inspecting the face, feeling for heaves and thrills and auscultation. Most of the checklist items missed by the candidate involve the auscultation steps. If we were to split the cardiovascular examination into steps to teach, we could have an introduction, general examination, precordial examination and auscultation step. For this candidate, they would benefit most if we focused our deconstructed component on the auscultation stage only.

In part 1 of the 3-D skills model the tutor will demonstrate the correct procedure for auscultating the chest only. Although other checklist items were missed, they will only focus on this part of the examination.

In part 2 of the 3-D skills model the tutor will guide the student as they perform the auscultation component only, prompting each step. The student does not repeat the rest of the examination.

In part 3 of the 3-D skills model the tutor encourages the student to repeat the corrected auscultation component, without prompting, but offering corrective coaching as required.
